# Impact and Challenges of Mesenchymal Stem Cells in Medicine: An Overview of the Current Knowledge

**DOI:** 10.1155/2018/5023925

**Published:** 2018-12-17

**Authors:** Farid Menaa, Somayeh Shahrokhi, V. Prasad Shastri

**Affiliations:** ^1^Department of Oncology, Stem Cells and Nanomedicine, California Innovations Corporation, La Jolla, San Diego, CA 92037, USA; ^2^Department of Immunology, Lorestan University of Medical Sciences, Khorramabad, Iran; ^3^Department of Cell Biology, University of Freiburg, Freiburg, Germany; ^4^Institute for Macromolecular Chemistry, Faculty of Chemistry and Pharmacy, BIOSS-Centre for Biological Signalling Studies, Stefan-Meier Strasse 31, Hermann Staudinger Haus, University of Freiburg, Freiburg D-79104, Germany

MSCs are multipotent adult SCs that exhibit the following main features [1–3]: (i) presence in almost any tissues (e.g., UBC, BM, WJ, skin, and dental); (ii) plastic adherence; (iii) capacity of self-renewing; (iv) ability of differentiating into multilineages (e.g., mesodermal (i.e., adipocytes, chondrocytes, and osteocytes) as well as ectodermal (e.g., neuronal cells) and endodermal (e.g., hepatocytes and pancreocytes)); (v) expression of specific set of cell surface markers according to the tissue origin (e.g., presence of CD73, CD90, and CD105 but absence of CD14, CD34, CD45, and HLA-DR); (vi) immunoregulatory properties (e.g., low alloreactivity and autoprotection from NKs due to low MHC I and lack of MHC II expressions along with costimulatory molecules such as CD80, CD40, and CD86; alleviation of disease response by favoring the conversion from Th2 (T helper cells) response to Th1 cellular immune response through modulation of IL-4 and IFN-*γ* levels in effector T-cells); (vii) homing capacity; and (viii) secretion of anti-inflammatory molecules (e.g., cytokines and receptors).

De facto, clinical applications of MSCs are tremendously promising in medicine (e.g., cell transplantation), pharmaceutical sciences (e.g., controlled drug delivery), and biological sciences (e.g., tissue engineering). They also provide greater advantages over other SCs (e.g., ESCs), which include [2, 4] (i) their relatively easy tissue isolation; (ii) the absence of obvious risk for the donor or ethical constraints; (iii) their capacity of migrating and homing to the injured site (e.g., tumor tropism) which can be tracked by noninvasive methods such as SPECT, BLI, or PET; (iv) their ability to expand for a relatively long period of time; (v) their ability to modify the host immune environment; (vi) their valuable immunomodulatory effects; and (vii) their higher *trans*differentiation potential as above specified.

Since the last 15 years, an increasing number of preclinical and clinical studies (>500) have been registered [5] using hMSCs as a valuable source in treatment of chronic diseases (e.g., autoimmune such as RA, inflammatory such as T1D, and CVD or degenerative diseases such as ALS, PD, and AD). Thereby, a number of studies [6, 7] pointed out the crucial role of MSCs in the improvement of RA, particularly at the onset of the disease, through a mechanism activating Treg cells and suppressing the production of inflammatory cytokines when injected into DBA/1 mice model. Also, transplantation of MSCs, when successfully differentiated into insulin-producing (beta) cells, was able to correct the hyperglycemia of STZ-induced diabetic rodents, enhance the survival rate of engrafted islets, and was found beneficial for treating non-insulin-dependent patients [8–10]. Besides, the transplantation of MSCs genetically modified to express GDNF improved the ALS phenotype and increased the number of neuromuscular connections [11]. In a pioneered study, MSCs delivered through nose to treat patients suffering from PD were found in different brain regions (e.g., hippocampus, olfactory lobe, and cortex) after 4.5 months of administration and could favorably modulate the expression of key enzymes (e.g., increased tyrosine hydroxylase and decreased toxin 6-hydroxydopamine levels) in the lesions of ipsilateral striatum and substantia nigra [12]. Moreover, MSCs were able to enhance the cell autophagy pathway, important in the amyloid plaque clearance, activated Tregs which in turn regulated microglia activation, and increased the neuronal survival both *in vitro* and in AD mice model [13, 14]. Eventually, transplantation of MSCs into myocardial infarction animal model along with fibronectin-immobilized PCL nanofibers was very successful [15].

Nevertheless, many of these clinical applications are hindered by research barriers [1, 16–21]. Remaining challenges, related to safety and efficacy of MSCs, include (i) the establishment of a comprehensive procedure for MSC isolation (e.g., methods may include Ficoll density gradient, collagenase, and marrow filter device) and for characterization/quality control (e.g., specific expression of cell surface markers, cell viability, endotoxin assays, and oncogenic tests); (ii) a proper setup of *in vitro* MSC expansion. Indeed, depending upon the severity of disease, an optimal dose of multipotent MSCs is required. The difficulty to obtain a large amount of adequate cells is often explained by the senescence manifested by shortening telomere length, decline in differentiation potential, and morphological alterations during a long-term *in vitro* culture under certain conditions which besides present the advantage to not favor spontaneous malignant transformation at higher passages (e.g., expansion of MSCs in controlled oxygen concentration and in serum-free culture media rather than supplemented with serum and/or growth factors); (iii) the cryopreservation and large-scale banking of clinical grade MSCs lack optimization in terms of medium to be used, uniformity in temperature during freezing and thawing, and storage time in liquid nitrogen. Interestingly, recent studies suggest that MSCs cryopreserved in serum-free culture media supplemented with CPAs (e.g., mixture of glucose, sucrose, and ethylene glycol in PBS and polyvinylpyrrolidone) can be successful to prevent any freezing damage to cells and toxicities related to the routine use of DMSO; (iv) a specific administration time and route (e.g., intravenous, in situ*/local*, and nasal) remains to be decided in order to fully maintain the functional capacity of a larger number of MSCs. In this regard, it is thought that the most convenient and feasible way of MSC transplantation is local injection to the site of injury or near the site of injury; (v) the underlying mechanisms that regulate and modulate these MSCs should be better understood. For instance, homing of MSCs involves CXCR4 and SDF-1 alpha but the exact mechanism is still unclear to avoid off-target homing; (vi) the precise mechanism(s) by which MSCs regulate the immune response is/are also undefined.

From the overall studies published to date, it becomes thus clearer that the use of hMSCs for clinical applications, at least in regenerative medicine, will increase.

## Abbreviations

AD: Alzheimer disease
ALS: Amyotrophic lateral sclerosis
BLI: Bioluminescence imaging
BM: Bone marrow
CD: Cluster of differentiation
CPAs: Cryoprotective agents
CVD: Cardiovascular diseases
DMSO: Dimethylsulfoxide
ESCs: Embryonic stem cells
GDNF: Glial cell line-derived neurotrophic factor
HLA: Human leukocyte antigen
IFN: Interferon
IL: Interleukin
MHC (I/II): Major histocompatibility complex (either class I or II)
MSCs: Mesenchymal stem cells
NKs: Natural killers
PBS: Phosphate-buffered saline
PCL: Polycaprolactone
PD: Parkinson disease
PET: Positron emission tomography
RA: Rheumatoid disease
SCs: Stem cells
SDF-1 alpha: Stromal cell-derived factor 1-alpha (or CXCL12)
SPECT: Single photon emission computed tomography
STZ: Streptozotocin
T1D: Type-1 diabetes
UCB: Umbilical cord blood
WJ: Wharton's jelly.
