# Corrigendum to “Impact and Challenges of Mesenchymal Stem Cells in Medicine: An Overview of the Current Knowledge”

**DOI:** 10.1155/2019/5493654

**Published:** 2019-03-10

**Authors:** Farid Menaa, Somayeh Shahrokhi, V. Prasad Shastri

**Affiliations:** ^1^Department of Oncology, Stem Cells, and Nanomedicine, California Innovations Corporation, La Jolla, San Diego, CA 92037, USA; ^2^Department of Immunology, Lorestan University of Medical Sciences, Khorramabad, Iran; ^3^Institute for Macromolecular Chemistry, Faculty of Chemistry and Pharmacy, BIOSS-Centre for Biological Signalling Studies, University of Freiburg, Hermann Staudinger Haus, Stefan-Meier Strasse 31, Freiburg D-79104, Germany

In the article titled “Impact and Challenges of Mesenchymal Stem Cells in Medicine: An Overview of the Current Knowledge” [1], there was an error in the affiliation details of the last author, as the affiliation “Department of Cell Biology, University of Freiburg, Freiburg, Germany,” was wrongly included.

The corrected authors' list and affiliations are shown above.
